# Autaptic Cultures: Methods and Applications

**DOI:** 10.3389/fnsyn.2020.00018

**Published:** 2020-04-30

**Authors:** John M. Bekkers

**Affiliations:** Eccles Institute of Neuroscience, The John Curtin School of Medical Research, The Australian National University, Canberra, ACT, Australia

**Keywords:** autapse, culture, methods, review, synapse

## Abstract

Neurons typically form daisy chains of synaptic connections with other neurons, but they can also form synapses with themselves. Although such self-synapses, or autapses, are comparatively rare *in vivo*, they are surprisingly common in dissociated neuronal cultures. At first glance, autapses in culture seem like a mere curiosity. However, by providing a simple model system in which a single recording electrode gives simultaneous access to the pre- and postsynaptic compartments, autaptic cultures have proven to be invaluable in facilitating important and elegant experiments in the area of synaptic neuroscience. Here, I provide detailed protocols for preparing and recording from autaptic cultures (also called micro-island or microdot cultures). Variations on the basic procedure are presented, as well as practical tips for optimizing the outcomes. I also illustrate the utility of autaptic cultures by reviewing the types of experiments that have used them over the past three decades. These examples serve to highlight the power and elegance of this simple model system, and will hopefully inspire new experiments for the interrogation of synaptic function.

## Introduction

The brain achieves its astonishing feats of information processing in part because of the complexity of its synaptic connections. Many synaptic circuit motifs have been elucidated, including feedforward, feedback, recurrent and lateral inhibition and excitation (Douglas and Martin, 2007; Yuste, 2015). Perhaps surprisingly, one of the most simple circuit motifs of all—that in which a neuron makes a synaptic connection with itself—was relatively late to come to the attention of neuroscientists. The term “autapse” entered the neuroscience lexicon only in 1972 when it was first coined to describe putative self-synapses on Golgi-stained pyramidal neurons in rabbit neocortex (Van der Loos and Glaser, 1972). Since then, both anatomical and physiological evidence for autapses *in vivo* has accumulated steadily (Karabelas and Purpura, 1980; Park et al., 1980; Peters and Proskauer, 1980; Preston et al., 1980; Lübke et al., 1996; Cobb et al., 1997; Tamás et al., 1997; Pouzat and Marty, 1998, 1999; Pawelzik et al., 2003; Bacci and Huguenard, 2006; Connelly and Lees, 2010; Manseau et al., 2010; Jiang et al., 2012, 2015; Yin et al., 2018; Deleuze et al., 2019). Today there is no question that autapses exist in the brain, albeit in much smaller numbers than (hetero-) synapses. However the importance of autapses for the normal operation of neural circuits remains a matter for speculation (Bekkers, 1998, 2003, 2009; White et al., 1998; Li et al., 2010; Connelly, 2014; Deleuze et al., 2014; Guo et al., 2016; Wiles et al., 2017).

In parallel with these discoveries about autapses in intact brain tissue, it was found that, under the right conditions, autapses in neuronal cultures are surprisingly common. When constrained to grow in isolation on “microislands” or “microdots” a few tens of microns across (Furshpan et al., 1976, 1986; Landis, 1976), cultured neurons readily form autapses (Segal and Furshpan, 1990; Bekkers and Stevens, 1991). The presence of two or more neurons on the microisland does not seem to curtail autapse formation (Tarsa and Goda, 2002; Wierda and Sørensen, 2014), suggesting that neurons are just as likely to form autapses as synapses when given the opportunity. It is possible that the 2-dimensional geometry of cultures, with the greater likelihood that an axon will encounter its dendrites, is an important reason why autapses are so prevalent in culture (Ikeda and Bekkers, 2006).

Although initially a curiosity, autaptic cultures have proven to be a valuable model system for addressing a range of important questions in cellular neuroscience. By providing a homogeneous population of synaptic contacts on a single, isolated neuron, autaptic cultures offer the ultimate in synaptic reductionism. Their functional simplicity has enabled many important and elegant experiments that would not have been possible in more complex systems.

The goals of this article are, first, to show how to prepare and utilize these cultures and, second, to give an overview of their many applications, with an emphasis on neurophysiological experiments. An alternative method for studying single neurons in isolation is to use very-low-density cultures, which have been described in detail elsewhere (e.g., Goslin et al., 1998; Ventimiglia and Lindsay, 1998; Fath et al., 2009); I will not be discussing this approach here. I will also not be discussing the use of similar cultures to study neurite growth on patterned substrates, which is of interest to the design of brain-machine interfaces (e.g., Jang et al., 2016; Gautam et al., 2017).

## Methods for Preparing Autaptic Cultures

Autaptic cultures are prepared in essentially the same way as conventional dissociated primary cultures (“mass cultures”), the main difference being the preparation of the culture plates. Several excellent articles about preparing autaptic cultures have been published (Segal et al., 1998; Allen, 2006; Fasano et al., 2008; Rost et al., 2010; Burgalossi et al., 2012; Lu et al., 2016) and the guidance below draws upon all of these, as well as my own experience (Bekkers and Stevens, 1991; Bekkers, 2005). It should be kept in mind that cell culture is often laced with superstition. The best advice is to start simple and elaborate only if necessary.

The methods presented here will focus on general-purpose hippocampal or cortical cultures prepared from newborn mice or rats. Others have described how to prepare cultures from embryos (Fath et al., 2009; Lu et al., 2016), older tissue (Ogata and Tatebayashi, 1991; Brewer, 1997; Allen, 2006) and other brain areas (Johnson, 1994; Shi and Rayport, 1994; Sulzer et al., 1998; Michel and Trudeau, 2000; Moechars et al., 2006). There is even a protocol for preparing autaptic cultures from human induced pluripotent stem cells (Fenske et al., 2019). Whatever the tissue source, it goes without saying that all procedures must be approved by the local ethics committee.

### Overview of the Procedure

**Figure 1:**
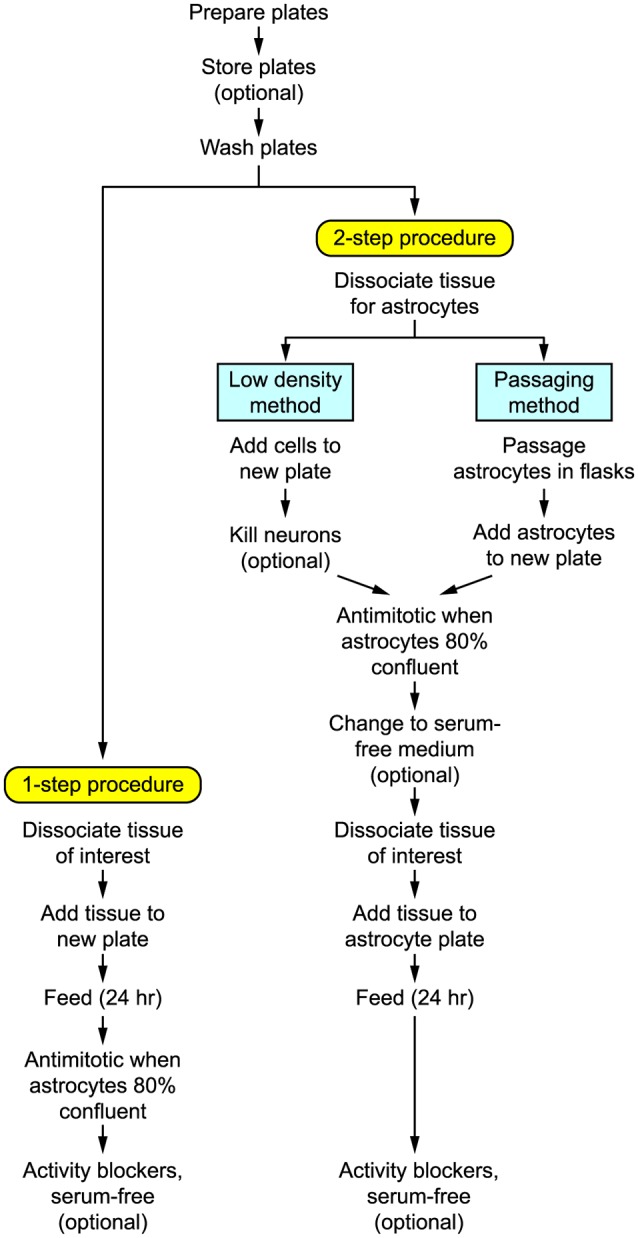
Flow diagram summarizing the steps involved in preparing autaptic cultures.

Figure 1 shows the basic steps in preparing autaptic cultures, and the detailed requirements are listed in Tables s 1–4. Briefly, the key initial step is to prepare coverslips with spots of permissive growth substrate (e.g., collagen, poly-D-lysine) dispersed across a coating of non-permissive substrate (agarose). Dissociated primary neurons are then added to the coverslips, usually (but not always) after first growing a monolayer of glial cells (astrocytes) on the spots to provide trophic support for the neurons. Autaptic neurons are typically ready for use after 1–2 weeks *in vitro*. The entire procedure will take at least 2–3 weeks, depending on the exact method used.

**Table 1:** Solutions for preparing plates.

Permissive coating solution
Stock solutions:
5 mg/ml poly-D-lysine in sterile dH_2_O Collagen as assayed by the supplier
To prepare 1 ml of permissive coating solution:
Aim for final concentrations of ~0.1 mg/ml poly-D-lysine and ~0.5 mg/ml collagen Dilute the collagen to 0.5 mg/ml in 1 ml of sterile dH_2_O Add 20 μl of poly-D-lysine stock to the 1 ml of diluted collagen
Keep the coating solution for several weeks at 4°C.
**Quantity**	**Name**	**Supplier**	**Catalog number**
5 mg	Poly-D-lysine hydrobromide 30-70K	Sigma–Aldrich	P7280
20 ml	Rat tail collagen	Thermo Fisher	A1048301
		Sigma–Aldrich	C3867
5 g	Agarose, Type II-A: medium EEO	Sigma–Aldrich	A9918

*Notes: 1. Poly-L-lysine, and either the hydrobromide or hydrochloride salt, should work as well, but some experimentation may be required. 2. Some investigators recommend preparing the poly-D/L-lysine stock in borate buffer (1.24 g of boric acid, 1.9 g of sodium tetraborate, 400 ml of H_2_O)*.

### Preparation of Plates

(1) *Clean the coverslips*. We use 24-well culture plates and grow the cells on 12–13 mm diameter No. 1 circular glass coverslips, one per well, which routinely provides 1–5 isolated single neurons per coverslip, i.e., up to this number of autapse experiments per well. Larger coverslips may be more convenient in some situations (see Step 5). Clean the coverslips by swirling them for 5 min in 10 N nitric or hydrochloric acid. This is intended to make the glass more hydrophilic for the agarose coating, but simply cleaning with ethanol also works (Lu et al., 2016). Rinse thoroughly in tap water, followed by distilled water then 100% ethanol, and store in a glass bottle under 100% ethanol. A large number can be cleaned at the same time and used for months.
(2) *Place coverslips in culture plates*. Using forceps and working in a sterile hood, flame the coverslips dry one at a time by passing them through a gas flame. Place the coverslips in the culture plates, one per well. Center the coverslip in each well and press down gently to “stick” it in position.
(3) *Coat coverslips with agarose*. Prepare 0.15–0.2% agarose in dH_2_O. This can be done by weighing 15–20 mg of agarose into 10 ml of dH_2_O in a 15 ml centrifuge tube. The tube, with its cap loosened, is microwaved or placed in boiling water for 2–5 min until the agarose is dissolved. Using a 200 μl pipette, spread a thin layer of the agarose solution on each coverslip then suck off the excess (It helps to first stick down the coverslip by placing a drop of solution at the edge of the coverslip so it runs underneath). It is important that the whole coverslip is covered with an agarose solution; any gaps can allow astrocytes or neurons to grow on the glass, compromising the required isolation of microislands. Sometimes the glass does not wet well; in this case, you need to add more agarose solution so a large drop completely covers the glass. Allow the plate to dry in the hood. We usually prepare 4 or 5 plates like this at once then store them at room temperature for several months (Burgalossi et al., 2012).
(4) *Prepare the permissive substrate*. Prepare about 1 ml of permissive substrate solution. We normally use a mixture of poly-D-lysine and rat tail collagen (Table 1) because cells attach better to poly-D-lysine (Segal et al., 1998) but collagen adds viscosity that may help with the spotting (next step); however, either could be used on its own. The mixture keeps for several weeks at 4°C. Possible alternatives are poly-L-lysine and poly-D,L-ornithine (Segal et al., 1998).
(5) *Apply spots of permissive substrate*. The next step—dispersing small spots of poly-D-lysine/collagen on the agarose coating—is critical. The original method uses a micro atomizer or airbrush to spray a mist of permissive substrate onto the agarose-coated coverslips, which requires judgment and experience to achieve a satisfactory distribution of small, discrete spots (Segal and Furshpan, 1990; Bekkers and Stevens, 1991; Fasano et al., 2008). A more recent method is to fabricate a stamping tool that imprints a regular pattern of permissive substrate solution onto the coverslips (Moulder et al., 2007; Sgro et al., 2011; Burgalossi et al., 2012; Ricoult et al., 2012).
(i) *Microatomizer* *method*. Obtain a small glass reagent sprayer or artist’s airbrush (see Table 4 for suggested sources). A small perfume bottle may also work. Sterilize the sprayer by spraying 80% ethanol and allowing it to dry in the culture hood. Load the substrate solution into the atomizer and spray the coating solution onto the dry agarose-coated plates. The spraying pressure, spraying duration, and distance from the plate need to be optimized so that small, discrete spots (~200 μm diameter) of the substrate solution settle on the coverslip. Here are some tips to guide mastery of this critical step (see also “Troubleshooting” section below).
• Hold the culture plate vertically (the coverslips should not fall out if they have been stuck down with agarose) against a dark surface for better visibility then apply a brief burst of spray from about 15 cm away to cover each half of the plate.
• Alternatively, place the plates on a horizontal surface and spray from ~50 cm away, allowing the mist to settle on the plates (Fasano et al., 2008).
• Higher pressure in the atomizer tends to produce smaller spots, but the spraying time needs to be brief to avoid producing too many spots that merge.
• The spattering can be practiced by spraying an uncoated coverslip with a test solution in which a dye (e.g., Trypan blue) is added to the normal coating solution (to have the right viscosity). The distribution and sizes of the spots can be observed under a dissecting scope immediately after spraying, then the spots can then be wiped off and another attempt made.
• The spots may vary markedly in size, which is acceptable; you only need ~10–20 spots on the coverslip that have the preferred size (~200 μm).
(ii) *Stamping tool* *method*. This method requires the fabrication of a special “microstamp” but yields much more reproducible microislands and, hence, a higher yield of isolated autaptic neurons (Moulder et al., 2007; Rost et al., 2010; Sgro et al., 2011; Burgalossi et al., 2012; Ricoult et al., 2012). The stamp can be made using photolithography and microcontact printing techniques. A typical pattern might be an array of squares or circles, 150–200 μm across, spaced at 400–500 μm intervals (Sgro et al., 2011; Burgalossi et al., 2012). The permissive substrate solution is loaded onto the stamp then transferred to the agarose-coated coverslips with gentle pressure. The key precaution with this method is to avoid under- or overloading the stamp with substrate solution (Moulder et al., 2007; Burgalossi et al., 2012).
(6) *Sterilize and store the prepared plates*. Allow the plates to dry in the hood, which should only take a few minutes. It is advisable to sterilize the plates by placing them under a UV lamp in the culture hood for 20–30 min. Prepared plates can then be covered and kept at room temperature for several weeks before use. Indeed, doing so has been reported to result in healthier cultures (Sgro et al., 2011), although others state that the plates should be prepared just before use (Fath et al., 2009).
(7) *Wash the plates*. The day before the cell preparation, add a few drops of culture medium to each well—just enough to cover the coverslip—and keep the plates in an incubator overnight. This “washes” the substrate and seems to improve the survival of cells. Just before adding the cell suspension to the plates the washing medium is sucked out and discarded.

**Table 2:** Solutions for tissue dissociation.

Dissection solution
This can be standard mammalian Ringer containing (in mM): 125 NaCl, 3 KCl, 2 CaCl_2_, 1 MgCl_2_, 25 HEPES @ pH 7.4 adjusted with NaOH, 10 glucose, 25 sorbitol to give 315 mOsm/kg, 0.22 μm filtered to sterilize.
**Enzyme solution**
Mix in a 15 ml tube:
5 ml Hank’s Balanced Salt Solution (BSS; divalent-free, with phenol red) 50 μl 50 mM EDTA stock 75 μl 100 mM CaCl_2_ ~1 mg cysteine 100 units papain suspension
Add about 10 μl of 1 M NaOH to adjust pH to about 7 (i.e., a pale pink color).
Keep in 37°C bath for 10–15 min to dissolve papain, then 0.22 μm filter into another sterile 15 ml tube and return the tube to the 37°C bath until required.
**Quantity**	**Name**	**Supplier**	**Catalog number**
500 ml	Hank’s BSS, divalent-free, with phenol red	Thermo Fisher	14170112
100 g	EDTA	Sigma–Aldrich	E6758
5 g	L-cysteine, HCl monohydrate	Sigma–Aldrich	C7889
100 mg	Papain, suspension	Worthington	LS 003126
		Sigma–Aldrich	P3125

**Table 3:** Culture media.

Classic serum-containing medium
To prepare 100 ml:
2 ml of 1 M stock glucose in MEM with Earle’s Salts 5 ml of heat-inactivated Fetal Bovine Serum (FBS) 1 ml of 5,000 units/ml stock Penicillin-Streptomycin MEM, Earle’s salts, top-up to 100 ml 100 μl Serum Extender
Filter (0.22 μm) into a sterile bottle. Keep in the dark at 4°C for about 2 weeks.
**Quantity**	**Name**	**Supplier**	**Catalog number**
1,000 ml	Minimal Essential Medium (MEM) with Earle’s Salts, without glutamine	Sigma–Aldrich	51412C
100 ml	FBS	Various
100 ml	Penicillin-Streptomycin	Thermo Fisher	15070063
1 vial	MITO+ Serum Extender	Corning	355006
**Alternative serum-containing medium**
To prepare 100 ml:
50 ml DMEM with HEPES: Weigh out 870 mg Dulbecco’s Modified Eagle’s Medium (DMEM) with high glucose (powder), add <50 ml distilled water (dH_2_O) Mix the 50 ml prepared in step 1 with 50 ml of DMEM with high glucose (liquid). 2 ml B-27 supplement. 1 ml of 5,000 units/ml stock Penicillin-Streptomycin. 5 ml heat-inactivated FBS. Add about 350 μl 1 M NaOH to adjust pH to about 7.5.
Filter (0.22 μm) into a sterile bottle. Keep in the dark at 4°C for about 2 weeks.
**Quantity**	**Name**	**Supplier**	**Catalog number**
For 10 L	Dulbecco’s Modified Eagle’s Medium with high glucose (powder)	Sigma–Aldrich	D1152
100 ml	Dulbecco’s Modified Eagle’s Medium with high glucose (liquid)	Sigma–Aldrich	D0422
10 ml	B-27 Supplement (50×)	Thermo Fisher	17504044
100 ml	Penicillin-Streptomycin	Thermo Fisher	15070063
100 ml	FBS	Various	
**Complete Neurobasal medium (serum-free)**
To prepare 100 ml, mix aseptically:
98 ml of Neurobasal or Neurobasal Plus medium 2 ml of B-27 or B-27 Plus Supplement 250 μl of GlutaMAX-I Supplement
Keep in the dark at 4°C for about 2 weeks.
**Quantity**	**Name**	**Supplier**	**Catalog number**
500 ml	Neurobasal Medium	Thermo Fisher	21103049
	Neurobasal Plus medium		A3582901
10 ml	B-27 Supplement (50×)	Thermo Fisher	17504044
	B-27 Plus Supplement (50×)		A3582801
100 ml	GlutaMAX-I Supplement	Thermo Fisher	A1286001

*Notes: 1. To heat-inactivate FBS, heat @ 56°C for 30 min in a water bath. 2. MEM without phenol red may be preferred for cultures that will be used for imaging experiments to minimize background fluorescence. 3. For suppressing the overgrowth of glial cells, prepare a 2.5 mM stock solution of cytosine arabinoside, free base (Sigma–Aldrich, C-1768) in dH_2_O and store the stock at −20°C. Dilute this in warmed culture medium to achieve a final concentration of 5 μM in the culture plate after feeding. 4. Glial cells grow more readily in medium supplemented with FBS, so an alternative for suppressing glial growth, particularly if plating onto an astrocyte feeder layer, is to replace the FBS with twice the concentration of horse serum*.

**Table 4:** Other requirements.

13 mm round glass coverslips, No. 1 (0.13–0.17 mm thickness), cleaned as described
24 well culture plates
56 mm sterile culture dish (for coarse dissection of tissue)
35 mm sterile culture dish (for fine dissection of tissue, if required)
15 ml sterile plastic centrifuge tubes (for preparing solutions, incubating in enzyme, trituration)
50 ml sterile plastic centrifuge tubes (for working aliquots of culture medium)
0.22 μm pore 25 mm diameter sterile syringe filters (for sterilizing solutions)
10 ml syringes (for use with syringe filters)
10 ml sterile pipettes (for dispensing medium and adding cell suspension to plates)
Sterile Pasteur pipettes plugged at the wide end with cotton wool (for dispensing solutions during dissection and doing trituration)
Hemocytometer and access to a microscope (for counting cells)
Dissection instruments: e.g., dissection scope, medium scissors for decapitation, small scissors for opening the skull, small flat spatula for removing the brain and doing coarse dissection, one pair fine forceps, one scalpel blade for mincing tissue
80% v/v ethanol in distilled water (for sterilizing the dissection instruments and work area)
Sterile bottles (for storing the culture medium and solutions used in the preparation)
Small atomizer (for spraying the culture plates). We have the most experience with a small glass reagent sprayer (e.g., Kimble 5 ml thin layer chromatography sprayer, kimble-chase.com) but a small recycled perfume spray bottle might work as well, or an artist’s airbrush (e.g., Aztec airbrush from www.testors.com). See “Preparation of Plates” section for more details.
Stamping tool for applying spots of permissive substrate. This is an alternative to the atomizer. The stamping tool yields more reproducible microislands but the tool needs to be fabricated. See “Preparation of Plates” section for more details.

*Notes: 1. Quantities depend on the number of cultures. We find that one newborn animal is sufficient to yield enough hippocampal neurons for one or two 24-well plates. 2. The composition of the glass used in the coverslips may affect the health of the cultures. Be prepared to test coverslips from different suppliers*.

A variation on the above is to use the “sandwich” method, in which the coverslip containing the neurons is opposed to and separate from an astrocyte feeder culture that provides diffusible growth factors to the medium (Brewer and Cotman, 1989). A simple way to prepare plates for this method is to simply scratch the bottom of each well with a needle (Lu et al., 2016). This raises small protuberances of plastic that will hold the coverslip just above the bottom of the well. Another method is to briefly touch the bottom of each well in three places with a hot soldering iron, raising small plastic welts. The coverslips for “sandwich” cultures are prepared by first distributing them in large (e.g., 60 mm) culture dishes. They are then coated with agarose and sprayed with a permissive substrate, as described above for the standard method. Finally, they are sterilized and stored in the large culture dishes until needed for the cell preparation. See “Alternative Plating Method” section below for further details.

### Plating Procedure

Broadly speaking, primary neuronal cultures can be prepared in two different ways (Figure 1):

(i) *One-step procedure*. Neural tissue is enzymatically dissociated and the single-cell suspension is plated at a high enough density that the neurons will survive while the astrocytes become sufficiently numerous to provide longer-term trophic support for the neurons. This method works best with rats or embryonic tissue.
(ii) *Two-step procedure*. A monolayer “lawn” of astrocytes without neurons is prepared first, then the neurons are plated on top of the astrocytes 1–3 weeks later. This method works well for both rat and mouse postnatal tissue and tends to give a more consistent yield of autaptic neurons (Pyott and Rosenmund, 2002). The astrocytic lawn can be prepared in two ways.
(a) *Low-density*: The dissociated cell suspension is directly added to the microisland plates at a low enough density that only the astrocytes survive.
(b) *Passaging*: The astrocytes are grown and passaged separately before adding them to the microisland plates.

For both the one-step and two-step procedures the cultures are more likely to be successful when prepared from embryonic or newborn tissue, e.g., from rodents aged between embryonic day 16 (E16) and postnatal days 0–3 (P0–3). If cultures need to be prepared from older animals, more elaborate procedures are required (Kay and Wong, 1986; Kaneda et al., 1988; Ogata and Tatebayashi, 1991; Brown et al., 1993; Magistretti et al., 1996; Brewer, 1997; Allen, 2006). The steps presented below assume that newborn rodent tissue is being used. Methods for using embryonic tissue are described elsewhere (Goslin et al., 1998; Fath et al., 2009).

Unless otherwise stated, all of the following steps are done in a sterile laminar flow hood.

(1) *Prepare instruments and solutions*. Assemble the dissection instruments (Table 4) in the hood and sterilize them with 80% ethanol. Prepare the enzyme solution (Table 2), chill the dissection solution (Table 2) and warm the culture medium (Table 3). Papain is most commonly used in the enzyme solution because it is regarded as gentler than trypsin for dissociating fragile neural tissue (Goslin et al., 1998). However, if preparing astrocytes for passaging (see Step 8 below) then trypsin is suitable and more economical. The choice of the culture medium is critical. For growing neurons under defined conditions, serum-free medium (e.g., Neurobasal medium; Table 3) is preferred. On the other hand, astrocytes grow better in serum-containing medium (Table 3, Rost et al., 2010; Burgalossi et al., 2012). Thus, unless the neurons are being plated onto a confluent monolayer of astrocytes (see Steps 6 and 8 below), it is advisable to start with a serum-containing medium to promote astrocyte division. If desired, the culture can be switched to a serum-free medium after the astrocytes have reached confluence. A disadvantage of using serum is that there may be batch-to-batch variability in the properties of the serum, and so it may be advisable to test small amounts of different batches before placing a larger order.
(2) *Extract the tissue*. One newborn rat or mouse pup provides enough hippocampal or cortical cells for one or two 24-well plates. Sacrifice the pup using an approved method of euthanasia and remove the brain into ice-cold dissection solution. Dissect out the hippocampi or cortical tissue, if possible peel off the meninges (enclosing membrane), then cut the tissue into roughly 1 mm square blocks.
(3) *Incubate the tissue in enzyme*. Using a sterile pipette, transfer the tissue pieces, with as little of the dissection solution as possible, to the sterile 15 ml centrifuge tube containing the enzyme solution. Place the tube in a 37°C water bath with agitator and leave it gently agitating for 30 min. Alternatively, gentle manual agitation for a few seconds every 10 min will suffice. While the tissue is incubating, prepare a plugged sterile glass Pasteur pipette by gently melting the tip of the pipette in a burner flame in the hood. You want to smoothen the sharp edges of the pipette tip without making the tip much narrower.
(4) *Dissociate the tissue*. At the end of the incubation, move the tube with tissue back to the laminar flow hood and (optionally) add 50–100 μl of 10 mg/ml DNAse I stock solution and wait for about 30 s. This breaks down DNA from damaged cells and reduces clumping during the subsequent trituration. Using the smoothed Pasteur pipette, suck off as much as possible of the supernatant enzyme solution and add 1–2 ml of warmed culture medium. After allowing the tissue to settle, suck off and discard the supernatant. Repeat this 3–4 times to completely wash out the enzyme. Add a milliliter or so of culture medium and very gently triturate the tissue 3–4 times. Wait for the pieces to settle, then save the supernatant (which should appear a little cloudy due to the suspension of dissociated cells) to another sterile 15 ml tube. Repeat this process 6–8 times, each time triturating slightly more vigorously (but still very gently) and saving the supernatant until the tissue is mostly dissociated. Ensure that no air bubbles get into the solution at any stage during this procedure. Being very gentle and careful is the key to success. You should finish with 6–10 ml of single-cell suspension.
(5) *Count the cells*. Counting is done using a hemocytometer on a phase-contrast microscope. A good preparation is one that contains many phase-bright neurons with the stumps of processes visible. However, we count all phase-bright cells, including ones without obvious processes. Typically we get a yield of about 15–25 × 10^4^ cells ml^−1^ from the CA1 regions of two hippocampi. Possible remedies for a low yield are given in the “Troubleshooting” section.
(6) *Determine the dilutions*. Table 5 gives suggested cell counts and volumes for different types of cultures. When using the two-step procedure we typically prepare two 24-well plates during each culture preparation: one in which we prepare a low-density astrocyte culture using a new culture plate (*C* or *D* in Table 5), and one in which we plate neurons on top of a low-density astrocyte culture that was prepared 2–3 weeks previously and which is now a confluent monolayer (*E* or *F* in Table 5). Note that, when plating on top of an astrocyte monolayer (*E* or *F*), the suspension of neurons is simply added to the 0.5 ml of the medium that is already in each well. The low-density astrocyte cultures (*C* and *D* in Table 5) typically grow to confluency over 2–3 weeks with little contamination from surviving neurons. However, if neurons do remain, they can usually be lysed by simply removing the plate from the incubator for 30–60 min. Alternatively, the neurons can be killed by adding 0.2–1 mM glutamate to the culture medium for several hours, then rinsing and refeeding with fresh culture medium (Segal et al., 1998; Harms et al., 2005). Note that cell plating densities are advisory, and experimentation will be required to find densities that suit your particular conditions. In general, higher plating density leads to more surviving neurons with greater synaptic connectivity (Ivenshitz and Segal, 2010), but this may be a disadvantage if the aim is to maximize the number of microdots occupied by a single neuron. Further comments on plating density are made in the sections “Troubleshooting” and “Electrophysiological Recordings From Autapses.”
(7) *Incubate the culture plates*. Place the culture plate(s) into an incubator at 37°C, 5% CO_2_. The neurons should settle and stick to the substrate within an hour or two. They should begin to extend processes within 24 h, or sooner if plated on astrocytes.
(8) *Passaging method for preparing astrocyte monolayers*. Passaging provides an ample supply of pure astrocytes that can be plated out on new culture dishes at a higher density than in Step 6 above, which means that a confluent astrocytic “lawn” will grow more quickly. On the other hand, this approach is more complex. Briefly, neural tissue is dissociated as in Steps 1–4 above, except that harsher enzymes (e.g., trypsin) and more vigorous trituration are used to favor the more hardy astrocytes over neurons. The cells are grown in culture flasks under conditions that suit astrocytes and are passaged until required (Ullian et al., 2001).

**Table 5:** Cell dilutions.

Type of culture	Final cell density (×10^4^ ml^-1^)	Volume per well (24-well plate; ml)
*A*: 1-step procedure, rat	7–9	0.5
*B*: 1-step procedure, mouse	12–15	0.5
*C*: 2-step procedure, rat, to prepare a low-density astrocyte culture	3–5	0.5
*D*: 2-step procedure, mouse, to prepare a low-density astrocyte culture	3–5	0.5
*E*: 2-step procedure, rat, plating on an astrocyte monolayer	6–10	0.1
*F*: 2-step procedure, mouse, plating on an astrocyte monolayer	10–15	0.1

### Feeding

Following the plating of neurons, it is normal for significant neuronal death and accumulation of debris to occur over the first 24 h. The debris should be removed as much as possible by feeding the culture the day after the dissociation. If using 24-well plates, this is done by removing 0.15 ml of the old medium from each well (i.e., about 1/3 the well volume) and adding the same quantity of fresh, warmed medium.

A general rule of thumb is that autaptic cultures do better with minimal feeding, say, once every 1–2 weeks using the 0.15 ml off/on protocol above. However, there are several important exceptions to this rule.

(i) *When astrocytes approach confluence (one-step procedure)*. About 4–5 days after plating with the one-step procedure the astrocytes should be about 80% confluent. To prevent overgrowth of the neurons by astrocytes, the cultures should be treated with an antimitotic drug, e.g., cytosine arabinoside (araC, 5 μM final concentration; see Table 3, Notes), administered using the one-third off/on method above. The cultures are then fed again 1–3 days later with drug-free medium. Both of these feeds, as well as subsequent ones, can be done using a serum-free medium if desired, provided the transition is done slowly to avoid shock to the neurons.
(ii) *When astrocytes approach confluence (two-step procedure, preparing an astrocyte culture)*. Again, treatment with antimitotic should be done when the astrocytes reach about 80% confluence. In this case, the switch to a serum-free medium could be made by exchanging all of the medium at once, taking advantage of the greater robustness of astrocytes. However, it is important not to change the medium too soon (e.g., <2 days) before neurons are plated onto the astrocytes; the astrocytes need to be given time to “condition” the medium with trophic factors that aid neuron survival. The antimitotic should be left in place until after the neuronal suspension is added, to prevent mitosis of the newly added astrocytes.
(iii) *When the culture medium becomes acidic*. If the medium contains phenol red, acidity is apparent as a shift to a pale yellow color, indicative of an accumulation of acidic waste products. This change is often seen in older, denser cultures. If this occurs, feeding should be done more frequently to minimize damage to the neurons.

Particularly in older cultures, it may help to add blockers of synaptic transmission to the culture medium to reduce excitotoxicity. For example, after 7–10 days *in vitro* we sometimes add kynurenic acid (1 mM final concentration in each well), CNQX or DNQX (10 μM) to inhibit non-NMDA ionotropic glutamate receptors, or D, L-APV (40 μM) to inhibit NMDA receptors. We find that cultures typically survive much better for longer periods (>3–4 weeks) in the presence of one of these blockers. However, it should be kept in mind that chronic blockade of excitatory synaptic transmission may affect synapse maturation (Murthy et al., 2001).

### Alternative Plating Method

The “sandwich” culture is an elegant method for exposing neurons to astrocyte-conditioned medium without physical contact with the astrocytes (Brewer and Cotman, 1989; Goslin et al., 1998; Lu et al., 2016). Because the autaptic neurons are resting on coated glass rather than an astrocytic lawn, the quality of the optics is improved, which is beneficial for certain experiments. In this method, dissociated cells are plated in wells that do not contain coverslips but which have small plastic protuberances on the bottom, as described in the “Preparation of Plates” section. After 1–2 weeks a confluent mixed neuron-glia feeder culture is obtained. At this time another dissociation is done and the isolated cells are plated onto coverslips that had previously been agarose-coated and sprayed in large culture dishes (see “Preparation of Plates” section and Goslin et al., 1998). These large culture dishes are placed in the incubator for a few hours to allow the neurons to settle and attach, then each coverslip is taken out, inverted, and placed neuron side down in each well in the plate containing the feeder culture. The plastic protuberances hold the coverslip and adherent neurons just above the glia, allowing access to secreted growth factors without direct contact.

### Troubleshooting

**Figure 2:**
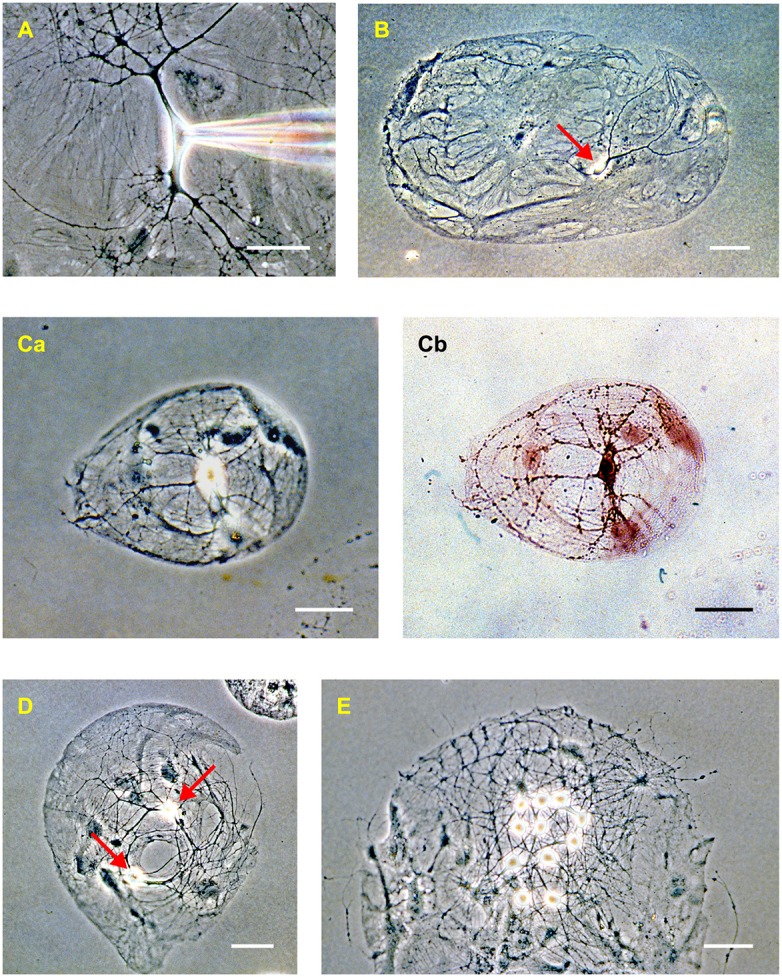
Rat hippocampal neurons in dissociated culture (2–3 weeks *in vitro*). **(A)** Phase-contrast image of an isolated neuron in a mass culture showing the phase-bright soma, thick tapering dendrites, and thin untapered axons. Confluent astrocytes are visible beneath the neuron. A patch electrode, entering from the right, is positioned on the soma. **(B)** “Microdot” or “microisland” of astrocytes occupied by a single neuron (red arrow). This is a comparatively large island. **(C)** A microisland of more typical size, occupied by a single neuron imaged using phase contrast **(Ca)** and bright-field **(Cb)** microscopy after processing with a synapsin antibody to reveal autapses (dark spots on the processes in **Cb**). **(D)** Example of a microisland occupied by two neurons (red arrows). **(E)** Example of a microisland with multiple neurons. The scale bar in all panels is 50 μm.

(1) *Agarose does not stick to the glass coverslips*. Make sure the coverslips have been thoroughly washed in concentrated acid and just as thoroughly rinsed in water. We have found that it helps to store the coverslips under 100% ethanol. If the ethanol contains too much water, the glass seems to be less easily wetted by agarose.
(2) *Problems with obtaining a suitable archipelago of microislands*. When using the spray technique, it is unnecessary to be too fussy about getting uniform spot sizes. It is normal to have a wide variety of sizes and shapes, provided that the spots are at least a few tens of microns apart and there is a reasonable number of spots (~10–20 per coverslip) that are roughly 200 μm in diameter. The challenge is to find the optimum parameter settings for the sprayer being used. Parameters to vary include airflow, spray burst duration, distance of sprayer from the coverslips, and size of the spray nozzle. For example, with our glass microatomizer (Kimble-Chase; see Table 4) we use airflow (from a reticulated air supply) at 3–4 L min^−1^, burst duration 0.5–1 s and spray distance ~10–15 cm.
(3) *Poor yield of cells following tissue dissociation*. The dissociation is the critical step for obtaining good autaptic cultures and, indeed, any dissociated cultures. Three factors seem key to achieving a good yield of healthy neurons, these being in the order of decreasing importance: (i) the age of the animal; (ii) the care in trituration; and (iii) the choice of dissociation enzyme. In general, the younger the animal the better the yield of neurons and, hence, the better the quality of the culture. Even a single day can make a difference: in our experience, P0 pups provide better neurons than P1 or P2 animals. For the best neuronal yield, embryos (e.g., E16–18) can be used, but this comes at the cost of more challenging surgery and the loss of the mother (Fath et al., 2009). Trituration is another important factor and requires you to be slow, gentle and patient. Each stroke of the trituration should, for example, take several seconds. The supernatant containing the cell suspension should be collected after every 3–4 strokes to avoid exposing isolated cells to unnecessary additional trauma. The tip of the Pasteur pipette used for trituration should be carefully fire-polished in the flame of a gas burner. Some researchers recommend using two Pasteur pipettes with wider and narrower polished openings; the narrower tip is used later in the trituration process (Fath et al., 2009). Finally, the type and activity of the enzyme are important. We find that papain is best for P0–1 rat tissue, but the suitability of alternative enzymes for other tissues (e.g., trypsin; Allen, 2006; Fath et al., 2009) needs to be determined empirically. Be aware also that the assayed activity of an enzyme can decline with time in storage.
(4) *Excessive neuronal death a few days after plating*. As noted in the “Feeding” section some loss of neurons is normal within a day or two of plating, particularly if not plating on astrocytes. It is important to remove the debris by feeding the day after plating because the debris seems to have an inhibitory effect on the survival of the remaining cells. Subsequent excessive neuronal death could be due to a multitude of factors that can be time-consuming to track down. For convenience, it is best to troubleshoot these factors using mass cultures rather than microdots. Three key factors to explore are: (i) the substrate; (ii) the astrocyte feeder layer; and (iii) the culture medium (Goslin et al., 1998). Substrate problems might include the properties or cleanliness of the glass coverslips, or the freshness of the permissive coating solution. These can be tested by plating directly on the coated or uncoated bottom of plastic culture dishes, which are generally optimized for good cell survival. If the neurons survive better on plastic, test the coating solution on different coverslips. Anecdotally, problems have been traced back to the type of glass used in different brands of coverslips, although this is less likely to be an issue for autaptic cultures where the glass is covered with agarose. If the neurons still die on plastic, they may require an astrocyte feeder layer, particularly if using mouse tissue or tissue from older (e.g., >P5) animals. Ideally, the astrocyte feeder layer should form a homogeneous extent of flat cells without too many gaps or overgrowth (e.g., Figure 2A). This is normally achieved by treating with an antimitotic agent (e.g., araC) as they approach confluence. The astrocyte cultures should also not be too old. We find that <3–4 weeks is preferred. Finally, the culture medium is probably the most important determinant of healthy cultures. Commercially available formulations are constantly evolving and some experimentation may be required to find the optimum for your application. Generally speaking, the serum-containing medium is better for growing astrocytes but it may be compromised by unpredictable variability between different lots of serum. Serum-free medium has the advantage of greater consistency but not all cell types may thrive in the absence of serum. Whatever the choice of medium, a universal precaution is that it should be fresh, sterile and adjusted to the correct pH and osmolarity.

## Electrophysiological Recordings from Autapses

In this section, I will give tips for performing the most basic kind of electrophysiology experiment with autaptic cultures, i.e., a whole-cell patch-clamp recording of autaptic current from a single, isolated neuron under physiological conditions. These techniques are readily extended to more elaborate experiments of the kind that are summarized in later sections.

### Choosing a Neuron

Broadly speaking, cultures can be used for electrophysiology experiments after ~7 days *in vitro* (DIV), although autaptic currents are expected to be small at that stage (Gomperts et al., 2000). Longer time in culture typically leads to a greater number of autaptic connections and larger currents per neuron (Gomperts et al., 2000), but there is also a progressive loss of neurons with culture age. We find that ~14–21 DIV is a good compromise for measuring large autaptic currents from healthy rodent neurons.

It is common to perform autaptic culture experiments in a HEPES-buffered bath solution at room temperature without bubbling or perfusion (e.g., Bekkers, 2005). Other standard bath solutions could also be used, for example, a perfused, carbogen-bubbled, bicarbonate-buffered bath solution warmed to physiological temperature, as in slice experiments (e.g., Ikeda et al., 2018). A phase-contrast microscope provides the best optics for observing neurites in culture (Figure 2). This may be important if one wishes to ensure, for example, that axons are not crossing the space between microislands. However, other types of contrast enhancement, such as differential interference contrast (DIC) or Hoffman modulation contrast, are acceptable for most studies with autaptic cultures.

After placing a whole coverslip in the chamber, the entire coverslip should be scanned at low magnification (e.g., 10× objective) to find potential microislands occupied by a single neuron. The shapes and sizes of these islands can be very diverse (Figure 2). In many cases it is unambiguous that a single neuron is present; in other cases, the identification is less clear. For example, glial overgrowth may obscure the view, or the soma of a glial cell (e.g., an oligodendrocyte) might be mistaken for that of a neuron. At other times the putative single neuron may occupy a very small island and it can be difficult to know if another neuron is hidden underneath the first. In these cases, the electrophysiology must be called upon to provide a definitive answer (see next section).

Identification of the type of neuron (e.g., glutamatergic, GABAergic, subtypes of these) can be inconclusive if simply based upon morphology. Dendritic morphology is often altered in culture, and in any case, the confinement of a neuron to a small island can make its dendrites hard to see. The best strategy is to prepare cultures from transgenic tissue in which the neurons of interest are fluorescently labeled (e.g., Ikeda et al., 2008).

During this initial scan, it is important to establish the health of the culture. The underlying layer of astrocytes should uniformly cover the islands of the permissive substrate, and the larger islands on the plate should be occupied by significant numbers of healthy-looking neurons (e.g., Figure 2E). There should be no sign of detachment of the astrocytes from the substrate, or of the agarose coating (which looks like a thin, transparent membrane) from the glass. If the culture contains clumps of many neuronal somata, perhaps extending thick fascicles of neurites between the clumps, then the plating density was too high. If there are healthy astrocytes but few neurons, then the plating density may have been too low.

Incidentally, cultures sometimes contain cells with vigorously waving cilia. These are ependymal cells from the meninges. They do not seem to interfere with experiments.

### Whole-Cell Patch-Clamp

**Figure 3:**
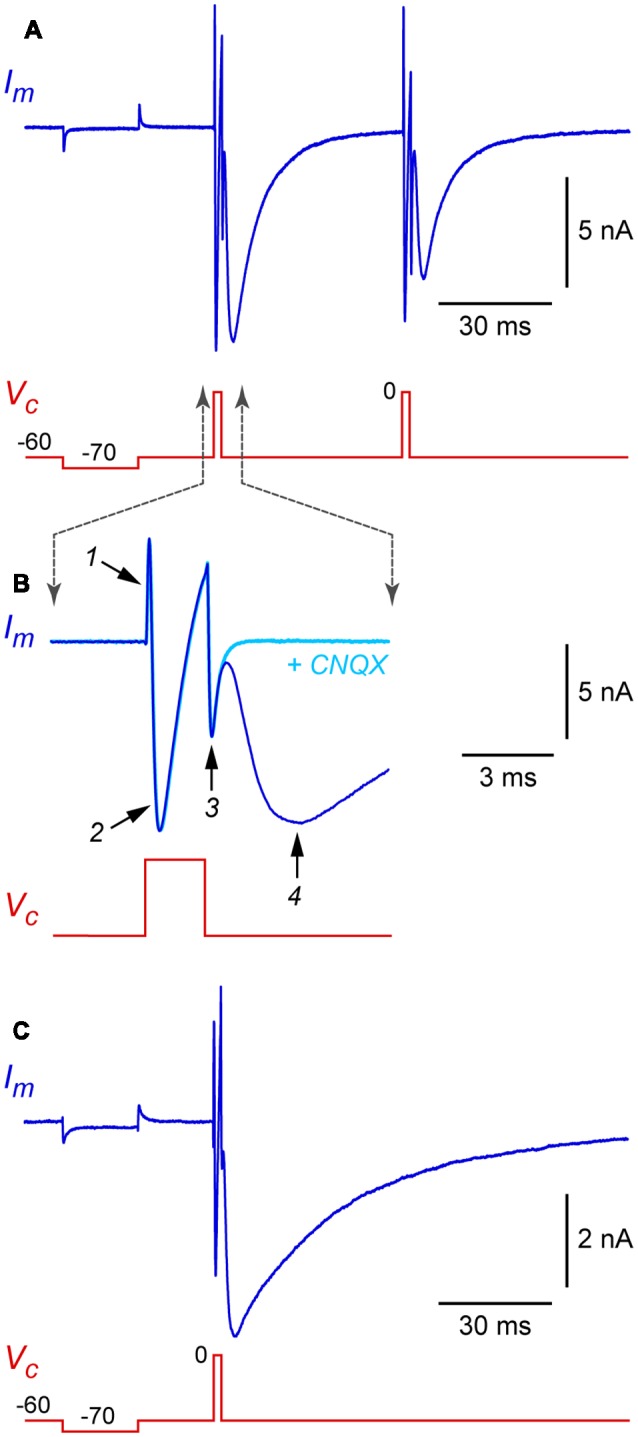
Example autaptic postsynaptic currents recorded from isolated rat hippocampal neurons. **(A)** Membrane current (*I_m_*, blue trace) recorded in response to a voltage clamp command (*V_c_*, red trace) in an isolated glutamatergic neuron. In this example, two depolarizing stimuli (each a 2 ms-long step from −60 mV holding potential to 0 mV) were applied at a 50 ms interval. A hyperpolarizing test pulse (20 ms-long step from −60 to −70 mV) preceded the depolarizing stimuli. **(B)** Same data shown on an expanded time scale to illustrate the phases of the *I_m_* response. Also shown (cyan trace) is *I_m_* from the same cell immediately after switching to a flow pipe containing 20 μM CNQX (6-cyano-7-nitroquinoxaline-2,3-dione) to block the excitatory postsynaptic current (EPSC). Labeled traces are (*1*) “on” capacitance transient, (*2*) action current (*I_Na_* and *I_K_*), (*3*) “off” capacitance transient, and (*4*) autaptic EPSC. **(C)** Similar recording from an isolated GABAergic interneuron, showing an autaptic inhibitory postsynaptic current (autaptic IPSC) in response to a single stimulus. Same time scale as in panel **(A)**; note the slower kinetics. In this experiment the pipette solution contained high Cl^−^, giving an inward current at −60 mV holding potential. Experiments in this figure were done at room temperature without series resistance compensation.

Standard patch-clamp techniques can be used with autaptic cultures, with a few precautions that are discussed below. After obtaining a stable whole-cell recording from the soma of an isolated neuron, the cell is voltage-clamped at a hyperpolarized holding potential (e.g., −60 mV) and a 1 ms-long depolarizing voltage-clamp step to 0 mV is applied (Figure 3A, this shows a hyperpolarizing test pulse followed by two depolarizing steps in succession). Each depolarizing step produces, in sequence, an outward capacitance transient (labeled *1* in Figure 3B), an inward “action current” (unclamped action potential; *2* in Figure 3B) and an inward capacitance transient (*3* in Figure 3B). The action current escapes into the axon as a propagating action potential and, if autapses are present, a voltage-clamped autaptic current appears after a delay (*4* in Figure 3B). In this example the neuron is glutamatergic and a fast inward excitatory autaptic current is observed. This autaptic current is selectively blocked by an inhibitor of AMPA receptors (Figure 3B, cyan trace). A similar experiment with an isolated GABAergic neuron shows an inhibitory autaptic current with slower kinetics, as expected for a current mediated by GABA_A_ receptors (Figure 3C; in this example the pipette solution contained high Cl^−^).

Experiments can also be performed in current-clamp mode, in which case autaptic potentials would be recorded (Bekkers and Stevens, 1991). However, voltage-clamp recordings are generally more convenient because the repeated firing of self-exciting neurons can generally be more effectively controlled; thus, most investigators use voltage clamp.

This basic autapse experiment illustrates some precautions to keep in mind when measuring autaptic currents. First, the internal solution should allow the neuron to fire brief action potentials, i.e., it should typically be a high-K^+^ solution to enable rapid spike repolarization. This is necessary to ensure the presynaptic action potential and the postsynaptic autaptic response are temporally dissociated, given that both are recorded in the same electrode (Figure 3). An internal solution that blocks K^+^ channels, such as a high-Cs^+^ solution of the kind commonly chosen to improve voltage clamp at depolarized potentials, should not be used.

A second precaution is that care must be taken to minimize voltage clamp errors. Autaptic currents can be large (up to 10 nA) and voltage escape could lead to uncontrolled action potentials and a reverberating feedback response in the neuron (Segal and Furshpan, 1990; Segal, 1991). Lower-resistance patch electrodes should generally be selected (e.g., <3–4 MΩ) to facilitate the achievement of low (<10–15 MΩ) electrode series resistance (*R_s_*). In addition to improving voltage control, low *R_s_* enables fast whole-cell capacitance transients (labeled *1* and *3* in Figure 3B) which are less likely to merge with and obscure the closely following autaptic current. If merging does occur and is a concern, a workaround is to pharmacologically isolate the transients and action current by briefly superfusing a blocker of postsynaptic receptors (e.g., CNQX for glutamatergic autapses; Figure 3B, cyan trace), then digitally subtracting the blocked trace from the unblocked trace. This procedure should yield the pure autaptic current with minimal contamination from transients. In any case, electronic series resistance compensation should generally be used when measuring autaptic currents to minimize voltage clamp errors (Williams, 2004). Some investigators further reduce voltage clamp errors by applying a low concentration of blocker of postsynaptic receptors to reduce the amplitude of the autaptic current (Otsu et al., 2004).

A related precaution is to ensure that *R_s_* remains as stable as possible throughout the experiment. It is common for *R_s_* to increase steadily over time, which destabilizes the *R_s_* compensation and causes an apparent rundown in the amplitude of the autaptic current. We have found that this increase in *R_s_* can be much reduced by using internal solutions with a higher osmolarity (e.g., 330 mOsm/kg *cf* 310 mOsm/kg for the external solution; Bekkers, 2005). Higher internal osmolarity causes the neuron to swell slightly, possibly reducing a tendency of intracellular organelles to block the electrode tip.

Finally, autaptic currents sometimes exhibit delayed peaks without a preceding action current. This is often a sign that there is more than one neuron on the microdot, giving rise to a polysynaptic circuit. For some kinds of experiments, the delayed polysynaptic currents may not be a concern, particularly if the early autaptic current dominates. Another strategy, if the second neuron is visible, is to destroy the first neuron using the patch electrode (e.g., by aspirating the soma) then lower a new electrode to record from the second neuron. This strategy is only useful if the presence on the second neuron of remnant synapses from the first neuron is not a concern.

## Applications of Autaptic Cultures

In this section, I will illustrate the utility of autaptic cultures by giving an overview of studies that have made use of them over the past three decades. I will organize my survey to highlight the unique technical advantages of autapses while paying less attention to the specific research questions being addressed. Most of the examples will be electrophysiology experiments using hippocampal and neocortical cultures, but some use autaptic cultures from other brain areas, including the thalamus (Moechars et al., 2006), basal forebrain (Allen, 2006), nucleus accumbens (Shi and Rayport, 1994), raphé nucleus (Johnson, 1994) and ventral tegmental area (Sulzer et al., 1998; Michel and Trudeau, 2000). First, I will make general comments about the advantages and disadvantages of autaptic cultures.

### Advantages

**Figure 4:**
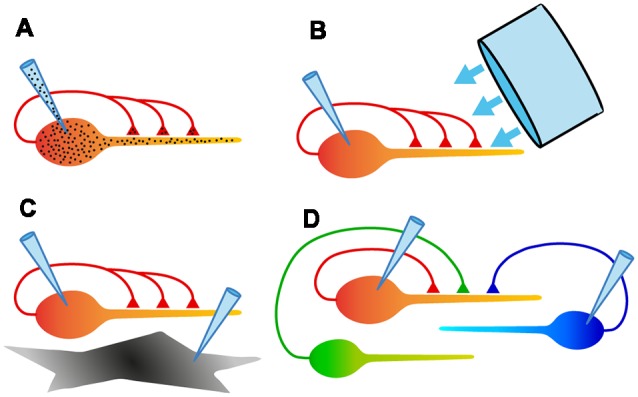
Schematic diagrams summarizing some of the experimental configurations possible with autaptic cultures. **(A)** Electrical recording from assured monosynaptic connections in a homogeneous set of autapses using a single electrode, and control of both pre- and postsynaptic intracellular solutions using a single electrode. **(B)** Ability to rapidly and uniformly apply extracellular solutions or light stimulation to all autaptic connections received by that neuron. **(C)** Study of signaling between a neuron and an underlying astrocyte, using two recording electrodes. **(D)** Study of competition between two or more neurons confined to a microisland. This also allows simultaneous comparison of synaptic and autaptic responses in the same neuron.

Like any dissociated culture model, autaptic cultures provide the convenience of a simplified two-dimensional structure: easy access to individual cells for making electrical recordings, and excellent visibility for imaging experiments. Also, however, autaptic cultures offer unique advantages (Figure 4).

(1) *The convenience of recording an unambiguous monosynaptic connection with a single electrode* (Figure 4A). Because of the ease of recording, autaptic cultures provide a simple assay system for studying factors that determine synaptic strength.
(2) *The ability to study a single axon and all its synaptic connections* (Figure 4A). A single axon means that the properties of a defined population of synaptic boutons can be studied, with no ambiguity about the origin of evoked or spontaneous synaptic events.
(3) *Access to a homogeneous population of modified synapses* (Figure 4A). Genetic modification of an isolated neuron ensures that all inputs onto the neuron are modified identically, providing an ideal testbed for structure-function studies.
(4) *Complete control over the extracellular environment of a neuron and all its synapses* (Figure 4B). Because all of their processes are restricted to a small area, autaptic cultures allow the rapid, homogeneous application of solutions or optical stimuli to an entire neuron, facilitating useful assays.
(5) *The ability to define the neighborhood of a neuron and study intercellular interactions* (Figures 4C,D). By selecting microislands occupied by different types or numbers of neurons or glia, interactions between these cell types can be explored in a simple system.

Examples of studies that explicitly draw upon these advantages are given in later sections.

### Disadvantages

Autaptic cultures are susceptible to disadvantages that apply to culture models in general. For instance, cultured neurons commonly lose the dendritic and axonal morphologies that characterize their appearance *in vivo*. Their intrinsic functional properties might also be modified, and this must be checked or stated as a proviso (see next section). In addition to these general concerns about cultures, however, autaptic cultures have some specific disadvantages. Because the same neuron provides both the presynaptic and postsynaptic compartment, it may be impossible to separate processes that occur both pre- and postsynaptically. For example, Ca^2+^ signals that originate from the presynaptic action potential and the postsynaptic response may be entangled because of dendritic backpropagation (Otsu and Murphy, 2004). It is also difficult to depolarize the postsynaptic membrane without affecting presynaptic excitability (Bekkers and Stevens, 1991). When performing whole-cell patch-clamp experiments, one must be aware of the possibility of “washout” of key intracellular components, which might be a greater concern for autaptic cultures because both pre- and postsynaptic components could be vulnerable. That said, stable autaptic responses can generally be obtained, provided the electrode series resistance and other recording conditions are carefully controled (see “Whole-Cell Patch-Clamp” section). Lastly, the setting up of autaptic cultures is more fickle and labor intensive than that of conventional cell cultures, chiefly because more elaborate substrate preparation is required.

### Comparison of Autapses With Synapses in Other Model Systems

As a prelude to discussing the applications of autaptic cultures, I will briefly comment on comparisons between the electrical properties of autapses and synapses in two other widely-used model systems, mass cultures, and acute brain slices.

#### Comparison With Interneuronal Synapses in Mass Cultures

The basic biophysical features of autapses and synapses in mass cultures are reported to be the same (Bekkers and Stevens, 1991) or similar (Shi and Rayport, 1994; Mennerick et al., 1995; Liu et al., 2009, 2013). Autaptic EPSCs have somewhat larger amplitudes and readily-releasable vesicle pool sizes than their mass-culture counterparts (Mennerick et al., 1995; Liu et al., 2013), and differences in short-term plasticity have also been reported (Liu et al., 2013). Overall, however, autapses and interneuronal synapses in culture appear to function similarly.

#### Comparison With Synapses in Acute Slices

Dissociated cultures—including autaptic cultures—are less able to preserve the synaptic “microenvironment” than are more intact preparations, like acute slices, and this raises questions about the wider applicability of findings made in the culture setting. Nevertheless, many experimental findings made with autapses have translated well to the intact brain. For example, detailed molecular studies of the synaptic vesicle cycle at autapses (see “A Homogeneous Population of Modified Synapses” section) have been confirmed more generally (Chamberland and Tóth, 2016; Dittman and Ryan, 2019). Certain types of synaptic plasticity that have been studied in slices are also faithfully replicated in autaptic cultures. For instance, depolarization-induced suppression of excitation (DSE; Straiker and Mackie, 2009) and inhibition (DSI; Straiker and Mackie, 2005; Kellogg et al., 2009) are present at autapses, as is long-term depression (LTD; Goda and Stevens, 1996; Tong et al., 1996; Kumura et al., 2000). Cultured dentate granule cells form autapses that resemble mossy fiber inputs to CA3 pyramidal cells, including expression of a presynaptic form of long-term potentiation (Rost et al., 2010). On the other hand, studies have also highlighted the potential risks of using cultures. For example, reports on the co-release of neurotransmitters (Hnasko and Edwards, 2012) and the efficacy of neuromodulators (Bekkers et al., 1996) show that the exact experimental conditions in culture can have an impact on the findings. All of this reinforces the point that, as for any model system, effective use of autaptic cultures must balance convenience and caution.

I will now turn to a survey of applications of autaptic cultures, organized under the five major advantages listed above in the “Advantages” section.

### Examples of Applications

#### The Convenience of a Single Electrode

By providing access to large, monosynaptic currents that can be stably measured with a single electrode, autaptic cultures have been used in many key experiments on the biophysics of synaptic transmission. Early examples include studies of multivesicular release at glutamatergic synapses (Tong and Jahr, 1994b), how transporters shape neurotransmission (Tong and Jahr, 1994a; Diamond and Jahr, 1997), and how the kinetics of EPSCs are determined by asynchronous vesicle release and temperature (Diamond and Jahr, 1995; Pyott and Rosenmund, 2002). The role of presynaptic Ca^2+^ in determining neurotransmission has also been examined using autapses (Reid et al., 1998; Lee et al., 2000; Ikeda et al., 2008).

A different application has been to use autaptic cultures to study neuromodulation, again taking advantage of their convenience as an assay system. Modulators that have been characterized with autapses include histamine (Bekkers, 1993), adrenoceptor agonists (Raman et al., 1996), calcineurin (Tong et al., 1995), somatostatin (Boehm and Betz, 1997), clozapine (Michel and Trudeau, 2000), cyclic AMP (Gekel and Neher, 2008), amyloid α protein (Ripoli et al., 2013) and GABA acting *via* GABA_B_ receptors (Valente et al., 2016). Several reports have also used autaptic cultures to examine short-term synaptic plasticity and its modulation (Mennerick and Zorumski, 1995a; Brody and Yue, 2000; Straiker and Mackie, 2007; Straiker et al., 2018). Lastly, autapses have been used as a convenient testbed for developing new molecular tools for the study of synaptic function (e.g., pHoenix, Rost et al., 2015).

#### A Single Axon

An autaptic neuron in culture is innervated by a single afferent (from itself) and thus provides access to a uniquely homogeneous population of presynaptic terminals—reliably stimulated—that can be used for the study of neurotransmitter release (Otsu and Murphy, 2004). For example, autaptic cultures have been utilized to measure the nonuniform probability of glutamate release from different boutons on the same axon (Rosenmund et al., 1993), the variable distribution of presynaptic Ca channel subtypes on a single afferent (Reid et al., 1997), and the differential invasion of glutamate-releasing terminals by action potentials (Prakriya and Mennerick, 2000). Autapses are also convenient for studying the synaptic vesicle cycle, for example, by using pharmacological approaches to count the number of releasable vesicles (Ikeda and Bekkers, 2009) or optical methods based on the styryl dyes (e.g., FM1–43) to follow vesicles through the cycle (Murthy et al., 1997; Murthy and Stevens, 1998). Experiments that load “false” neurotransmitters into synaptic vesicles have also been used to probe vesicle dynamics at autapses (Pan et al., 1993; Bekkers, 2005).

Another kind of experiment that relies on having a single axon takes advantage of the fact that both evoked and spontaneous postsynaptic currents originate from the same population of autaptic terminals, removing the ambiguity that typifies other kinds of synaptic circuits. For example, autaptic cultures been used to identify presynaptically silent synapses that give spontaneous but not evoked transmitter release (Kimura et al., 1997), and postsynaptically silent synapses that express only NMDA receptors (Gomperts et al., 1998). Autapses also allow the unambiguous study of phasic and asynchronous release from the same population of boutons (Otsu et al., 2004; Chang and Mennerick, 2010).

Lastly, the presence of a single axon enables definitive experiments showing the co-release of neurotransmitters from one neuron. For instance, autaptic cultures have been used to demonstrate the co-release of glutamate and serotonin from raphé neurons (Johnson, 1994) and of glutamate and dopamine from neurons in the ventral tegmental area (Sulzer et al., 1998).

#### A Homogeneous Population of Modified Synapses

Perhaps the most widely used application of autaptic cultures has been in structure-function studies, which take advantage of the ability to genetically modify a single isolated neuron and to examine the consequences of that modification for synaptic transmission, secure in the knowledge that all autapses received by that neuron are identically modified. The use of this culture system also enables the study of mutations that are postnatal lethal.

Genetically-modified autaptic cultures have been used to answer fundamental questions about the SNARE proteins, including SNAP-25 and SNAP-23 (Delgado-Martínez et al., 2007; Weber et al., 2014), syntaxin-1 (Gerber et al., 2008) and synaptobrevin-1 (Young, 2005; Zimmermann et al., 2014). Key experiments on the presynaptic Ca^2+^ sensor, synaptotagmin, have also been performed using autapses. For example, mutational analyses of synaptotagmin-1 (Fernández-Chacón et al., 2001; Stevens and Sullivan, 2003; Han et al., 2004; Nishiki and Augustine, 2004; Schupp et al., 2016; Chang et al., 2018) and synaptotagmin-4 (Ting et al., 2006) have elaborated details of the Ca^2+^ binding and synaptic vesicle fusion processes. The roles of many other presynaptic auxiliary proteins have been explored at autapses, including Munc13 (Augustin et al., 1999; Rosenmund et al., 2002; Varoqueaux et al., 2002; Junge et al., 2004; Basu et al., 2007), Munc18 (Meijer et al., 2015), synapsin (Gitler et al., 2004, 2008; Baldelli et al., 2007), bassoon (Altrock et al., 2003), synaptic vesicle protein 2 (Custer et al., 2006), RIM1α (Calakos et al., 2004), snapin (Thakur et al., 2004) and Rab3 (Schlüter et al., 2004). A number of these experiments took advantage of another convenient feature of autaptic cultures—the ability to completely superfuse a single neuron—to look for changes in the size of the readily-releasable pool (RRP) of synaptic vesicles. This feature will be further discussed in the next section.

Other researchers have used autapses and molecular genetic approaches to study the involvement of vesicular glutamate transporters in synaptic transmission (Fremeau et al., 2004; Wojcik et al., 2004; Zimmermann et al., 2015), as well as the properties of presynaptic voltage-gated Ca^2+^ channels (Few et al., 2012; Nanou et al., 2016). Lastly, a variety of disease models have been explored using this strategy. For example, genetic models of Alzheimer’s disease (Pratt et al., 2011), autism (Weston et al., 2014) and epilepsy (Heeroma et al., 2009) have been implemented and studied in autaptic cultures.

#### Control Over the Extracellular Environment

The ability to uniformly perfuse extracellular solution over an isolated autaptic neuron brings obvious advantages in pharmacological experiments, like the neuromodulation studies mentioned earlier (“The Convenience of a Single Electrode” section). However, uniform perfusion also enables other kinds of experiments that are difficult or impossible to achieve using other systems. Two main types of such experiments have been performed: measurements of synaptic vesicle pools using the application of hypertonic sucrose solution, and studies that disentangle subsynaptic (strictly, “sub-autaptic”) and extrasynaptic receptors by exploiting the unique configuration of autaptic cultures.

Sucrose experiments depend on the classical finding that hypertonic solution causes the direct release of synaptic vesicles without electrical stimulation (Fatt and Katz, 1952). By applying pulses of sucrose solution to the entire dendritic arbor of an isolated neuron on a small island, all presynaptic vesicle pools can be identically stimulated with a hypertonic challenge and the resulting postsynaptic electrical response can be recorded at the soma. Critically, spontaneous or electrically-evoked release from those same pools can also be recorded, allowing a comparison between the postsynaptic responses to the different release mechanisms. For example, by measuring the total charge carried by the (multi-vesicle) sucrose-evoked response, then dividing this by the mean charge of the (single-vesicle) spontaneous “miniature” synaptic response measured in the same neuron, one can estimate the total size of the RRP of synaptic vesicles across all release sites on that neuron (Rosenmund and Stevens, 1996; Stevens and Williams, 2007).

This method has been widely used to measure changes in the RRP size following genetic mutations, as mentioned in the previous section (“A Homogeneous Population of Modified Synapses”). The approach has also been used to study changes in RRP size following different kinds of plasticity, for example, long-term depression (Goda and Stevens, 1998), short-term depression (Stevens and Wesseling, 1998, 1999b) and augmentation (Stevens and Wesseling, 1999a; Garcia-Perez and Wesseling, 2008), as well as other factors that alter the RRP size at autapses (Stevens and Sullivan, 1998; Moulder et al., 2004; Moulder and Mennerick, 2005).

The second main type of experiment that exploits the ease of autapse superfusion is a method for distinguishing subsynaptic and extrasynaptic NMDA receptors. This experiment depends on the fact that electrical stimulation (e.g., *via* a whole-cell electrode at the soma) selectively activates subsynaptic NMDA receptors, whereas superfusing the entire neuron with glutamate activates both sub- and extrasynaptic NMDA receptors. Hence, by comparing these two responses one can infer the distinct properties of both pools of NMDA receptors (Tovar and Westbrook, 1999). In an elegant variation on this experiment, subsynaptic NMDA receptors are “tagged” by blocking them with an activity-dependent antagonist (MK-801) while electrically stimulating at the soma; the subsequent response to whole-cell application of glutamate must then be entirely due to unblocked extrasynaptic NMDA receptors (Tovar and Westbrook, 2002).

This approach has been used in several interesting studies. For example, the mobility and turnover of the different pools of NMDA receptors have been measured (Li et al., 2002; Tovar and Westbrook, 2002), as well as their subunit composition (Tovar and Westbrook, 1999; Thomas et al., 2006), pharmacology (Xia et al., 2010; Vyklicky et al., 2016) and desensitization properties (Li et al., 2003).

Lastly, the compact dendritic arbor of autaptic cultures facilitates the use of optical techniques for the study of synaptic function. For example, autaptic cultures permit the uniform elevation of intracellular Ca^2+^ in calcium uncaging experiments (Burgalossi et al., 2012). However, optogenetic techniques have not been used with autaptic cultures and provide an opportunity for interesting experiments in the future.

#### A Defined Neighborhood

The applications described so far have highlighted the isolation of autaptic neurons, but this final application takes advantage of microislands as a useful model for studying intercellular interactions in the local neighborhood. These experiments fall into two broad classes: signaling between an autaptic neuron and the underlying glial cells, and competition between neurons on microislands occupied by two or more neurons.

Neuron/glia signaling has been studied by making dual whole-cell recordings from an isolated autaptic neuron and an underlying glial cell (Mennerick and Zorumski, 1994, 1995a; Mennerick et al., 1996). These experiments have shown, for example, how transporters in the glia modulate the timecourse of the autaptic EPSC in the neuron (Mennerick and Zorumski, 1995b; Mennerick et al., 1999), and how autaptic neurons grown without glial contact exhibit more asynchronous neurotransmitter release (Sobieski et al., 2015). Culturing without contact with glia has also been shown to reduce the number of autapses that form (Ullian et al., 2001).

For studying interactions between neurons, microislands with two or more neurons are selected (Figure 4D). Early work used microislands with small numbers of neurons to study epileptiform activity, which tends to be very strong when neurons are spatially constrained in this way (Segal and Furshpan, 1990; Segal, 1991, 1994). More recent work, however, has tended to focus on two-neuron microislands for “mix and match” experiments. For example, it has been shown that chronically silencing one of a pair of isolated excitatory neurons leads to characteristic changes in the postsynaptic AMPA receptor subunit composition in each neuron (Harms et al., 2005). Other work has explored the competition between glutamatergic and GABAergic neurons when grown as mixed or homotypic pairs (Rao et al., 2000; Chang et al., 2014; Wierda and Sørensen, 2014), or between wildtype and mutant neurons that lack presynaptic proteins involved in transmitter release (Tarsa and Goda, 2002; García-Pérez et al., 2015). Finally, two-cell microislands with different combinations of striatal, cortical and thalamic neurons have been used to examine the effect of glutamatergic input on refining striatal output (Paraskevopoulou et al., 2019).

## Conclusion

Autaptic cultures provide the ultimate in synaptic simplicity: a one-neuron circuit. Despite a current tendency to study complex and preferably *in vivo* neural networks, autaptic cultures still have an important place in the toolbox of cellular neuroscientists because they enable straightforward, elegant experiments that address fundamental questions about synaptic transmission. The examples of applications given in this review provide a taste of the diversity of experiments that are possible with autaptic cultures, and will hopefully inspire novel applications, perhaps involving new-generation optical technologies. Of course, like all model systems, autaptic cultures have advantages and disadvantages, and these must be carefully weighed up when deciding whether autapses are the right tool for the job. It is hoped that this review has provided enough information to make that decision easier, and will assist in the implementation of autaptic culture experiments with the minimum pain and maximum prospect of success.

## Data Availability Statement

The datasets generated for this study are available on request to the corresponding author.

## Author Contributions

JB wrote the manuscript and prepared the figures.

## Conflict of Interest

The author declares that the research was conducted in the absence of any commercial or financial relationships that could be construed as a potential conflict of interest.
